# Pro-Inflammatory Response of Bovine Polymorphonuclear Cells Induced by *Mycoplasma mycoides* subsp. *mycoides*

**DOI:** 10.3389/fvets.2020.00142

**Published:** 2020-03-27

**Authors:** Marta Di Federico, Massimo Ancora, Mirella Luciani, Ivanka Krasteva, Flavio Sacchini, Gianluca Orsini, Tiziana Di Febo, Valeria Di Lollo, Mauro Mattioli, Massimo Scacchia, Giuseppe Marruchella, Cesare Cammà

**Affiliations:** ^1^Unit of Basic and Applied Biosciences, Faculty of Biosciences and Technology for Food, Agriculture and Environmental, University of Teramo, Teramo, Italy; ^2^Molecular Biology and Genomic Unit, Istituto Zooprofilattico Sperimentale dell'Abruzzo e del Molise “G. Caporale”, Teramo, Italy; ^3^Immunology and Serology Department, Istituto Zooprofilattico Sperimentale dell'Abruzzo e del Molise “G. Caporale”, Teramo, Italy; ^4^Bacterial Vaccines and Diagnostics Department, Istituto Zooprofilattico Sperimentale dell'Abruzzo e del Molise “G. Caporale”, Teramo, Italy; ^5^Cooperation Office, Istituto Zooprofilattico Sperimentale dell'Abruzzo e del Molise “G. Caporale”, Teramo, Italy; ^6^Faculty of Veterinary Medicine, University of Teramo, Teramo, Italy

**Keywords:** *Mycoplasma mycoides* subsp. *mycoides*, contagious bovine pleuropneumonia, polymorphonuclear cells, inflammatory mediators, gene expression

## Abstract

*Mycoplasma mycoides* subsp. *mycoides* (*Mmm*) is the etiological agent of contagious bovine pleuropneumonia (CBPP), one of the major diseases affecting cattle in sub-Saharan Africa. Some evidences suggest that the immune system of the host (cattle) plays an important role in the pathogenic mechanism of CBPP, but the factors involved in the process remain largely unknown. The present study aimed to investigate the cell response of bovine polymorphonuclear neutrophils (PMNs) after *Mmm in vitro* exposure using one step RT-qPCR and Western blotting. Data obtained indicate that gene and protein expression levels of some pro-inflammatory factors already change upon 30 min of PMNs exposure to *Mmm*. Of note, mRNA expression level in *Mmm* exposed PMNs increased in a time-dependent manner and for all time points investigated; targets expression was also detected by Western blotting in *Mmm* exposed PMNs only. These data demonstrate that when bovine PMN cells are triggered by *Mmm*, they undergo molecular changes, upregulating mRNA and protein expression of specific pro-inflammatory factors. These results provide additional information on host-pathogen interaction during CBPP infection.

## Introduction

*Mycoplasma mycoides* subsp. *mycoides* (*Mmm*) is the etiological agent of contagious bovine pleuropneumonia (CBPP), a severe respiratory disease of cattle notifiable to the World Organization for Animal Health (Office International des Epizooties [OIE]) (1). CBPP has been eradicated in most countries worldwide (http://www.oie.int/en/animal-health-in-the-world/official-disease-status/cbpp/en-cbpp) but remains widespread in sub-Saharan Africa, where it strongly impacts livestock productivity, draining financial resources to Governments for high cost of the control measures (2, 3).

*Mmm* belongs to mycoplasmas, the smallest wall-less and self-replicating microorganisms. So far, no typical virulence factors have been detected for *Mmm* and its virulence is probably multifactorial (4, 5). Some evidences suggest that the production of reactive oxygen species (ROS) (6, 7) and other immune-driven mechanisms (8) could contribute to CBPP lung injury but the factors promoting and sustaining those processes remain largely unknown.

Polymorphonuclear cells (PMNs) are the first line of cellular defense against invading pathogens, playing a critical role in innate immunity and influencing adaptive immune responses (9–11). In adult cattle PMNs represent the second most abundant leukocyte population with a neutrophil-to-lymphocyte *ratio* of ~1:2, which is lower compared to other domestic animals where PMNs represent up to 75% of the population of circulating leukocytes (12, 13). These cells are rapidly recruited to inflammatory and infection sites to provide early defense against invading microorganisms. At respiratory level, PMNs are among the major innate immune effector cells recruited during acute inflammation (14). PMNs are professional phagocytes and take part in pathogen clearance through several mechanisms like degranulation, phagocytosis, antibody derived cell cytotoxicity, and release of neutrophil extracellular traps (NETs) (15, 16). Beside their involvement in primary host defense against infections, PMNs also contribute to regulate inflammatory and immune responses (17). However, dysregulation of inflammatory stimuli leading to excessive neutrophils recruitment and activation may contribute to tissue injury (18). In CBPP, lungs showing acute-to-subacute stages of infection are characterized by an abundant cell inflammatory infiltrate containing PMNs and alveolar macrophages, suggesting the possible involvement of these cells in defensive and pathological mechanisms (19, 20).

Previous *in vitro* studies investigated the role of PMNs in the mechanism of interaction between cattle PMNs and *M. bovis* (21, 22) but few data are available on the interaction between *Mmm* and PMNs (7). Thus, this study aimed to investigate *in vitro* the expression of pro-inflammatory cytokines and inflammatory mediators induced in PMNs after *Mmm* exposure.

## Materials and Methods

### *Mycoplasma mycoides* subsp. *mycoides* Strain

Experiments were conducted using *Mmm* “Caprivi,” a highly virulent African strain available at the OIE Reference Laboratory for CBPP in Teramo (Italy), isolated in Namibia in 2003 (23). Cultures were grown in modified PPLO broth at 37°C in a 5% CO_2_ atmosphere for 2 days, then sub cultured and expanded to log phase for additional 44–48 h. Bacterial cells were obtained by centrifugation at 9,000 × g at 4°C for 40 min followed by two washes with isotonic phosphate-buffered saline (PBS, pH 7.2). Bacteria were re-suspended in PBS at a cell density of 10^8^ *per* ml.

### Isolation of Bovine PMNs and Exposure to *Mmm*

Blood samples were collected in EDTA containing tubes from clinically healthy and regularly slaughtered cattle (*n* = 3), selected in a CBPP free area (Italy). PMNs were isolated by density gradient using Ficoll Paque Plus (Merck KGaA, Darmstadt, Germany), according to manufacturer's instructions. Cell precipitate was treated with a hypotonic lysis buffer (155 mM NH4Cl, 10 mM KHCO3, 0.12 mM EDTA) to remove red blood cells. Then, PMNs were re-suspended in RPMI media (Merck KGaA, Darmstadt) to a concentration of 10^6^ *per* ml with a viability ≥ 90%, which was determined using an automated cell analyser (Vi-Cell, Beckman Coulter).

Re-suspended PMNs were seeded at a density of 5 × 10^5^ cells *per* well (500 μl) in 24-well flat-bottom plates (Falcon, Corning incorporated) with or without *Mmm* (5 × 10^7^cells *per* well) (500 μl) to obtain a multiplicity of infection (MOI) of 100. The plates were incubated at 37°C in 5% CO_2_ in mild shaking and cell suspensions were sampled at 30 min and 1, 2, 3, 6, and 18 h after *Mmm* exposure. Each sample (exposed PMNs and not exposed PMNs) was assessed in duplicate for every time point considered.

### RNA Extraction and RT-qPCR Analysis

After incubation in the absence or presence of *Mmm*, PMNs were pelleted, and RNA from the pellet was extracted using Direct Zoll RNA Kit (Zymo Research), which included a DNA digestion step (DNase I). Total RNA was quantified by Qubit RNA HS (High Sensitivity) Assay Kit (Thermo Fischer Scientific).

One step RT-qPCR assays were developed to quantify the relative expression of a panel of 7 target genes (interleukin-1β, IL-1β;interleukin 8, IL8; 5-lipoxygenase, 5-LOX; cyclooxygenase-2, COX-2; inducible nitric oxide synthase, iNOS; toll-like receptor 4, TLR4; tumor necrosis factor α, TNFα) involved in the inflammatory process. Primers and TaqMan probes (Eurofins Genomics) targeting IL8, TNFα (24), and COX-2 (25) were used as previously described in literature, while primers and probes for the other target genes were designed using Primer Express software (Applied Biosystem) (Table 1). RT-qPCR assay for each considered target was optimized and validated using lung sampled from CBPP infected cattle.

**Table 1:** Sequence of PCR primers and TaqMan probes.

**Target gene**	**Primer**	**Sequence (5′-35′)**	**Probe**	**Probe sequence (5′-3′)**	**Accession number**
β-ACT	ACT-F	CAGCACAATGAAGATCAAGATCATC	ACT-1081-Probe	TCGCTGTCCACCTTCCAGCAGATGT	AY141970
	ACT-R	CGGACTCATCGTACTCCTGCTT			
^*^COX-2	COX_2_Fw	CCAGAGCTGCTTTTCAACCAA	COX_2_Probe	TCCAGTACCAGAACCGT	AF031698
	COX_2_Rev	AGCGTGTTAAACTCAGCAGCAA			
IL1-β	IL1B_F	ACCTTCATTGCCCAGGTTTCT	IL1B_Probe	CAACCGTACCTGAACCCATCAACGAAA	EU276067
	IL1B_R	ACAGCTCATTCTCGTCACTGTAGTAAG			
^**^IL8	IL8.177F	CACTGTGAAAAATTCAGAAATCA*TT*GTTA	IL8.214P	AATGGAAACGAGGTCTGCTTAAACCCCAAG	S74436
	IL8.282R	CTTCACCAAATACCTGCACAACCTTC			
5-LOX	5ALOX_Fw	GAGATGGGCAAGCGAAGTTG	5ALOX_P	ACCAAATTCACGTTCTCAAGCAGCACAGA	NM_001192792
	5ALOX_Rev	TTTTGCCGTGTCTCCAGTTCT			
TLR4	TLR4_F	TGCGTACAGGTTGTTCCTAACATT	TLR4_Probe	AAAATCCCCGACAACATCCCCATATCAA	KX138607
	TLR4_R	CTGGAGAAGTTATGGCTGCCTAA			
iNOS	iNOS_Fw	TCTGCAGACACGTGCGTTATG	iNOS_P	ACAACGGCAACATCAGGTCGGCC	AJ699400
	iNOS_Rev	TCCAGACCCGGAAGTCATG			
^**^TNFα	TNF.338F	TCTTCTCAAGCCTCAAGTAACAAGT	TNF.367P	AGCCCACGTTGTAGCCGACATCAACTCC	Z14137
	TNF.440R	CCATGAGGGCATTGGCATAC			

* *Kanefsky et al. (25)*.

** *Leutenegger et al. (24)*.

RT-qPCR analysis were performed using SuperScript™ III Platinum™ One-Step qRT-PCR Kit (Invitrogen) according to manufacturer's instructions. The retro transcription and amplification were carried out in 96 well-plates employing 5 μl of RNA suspension in a 20 μl of reaction volume using QuantStudio 7 Flex Real-Time PCR System instrument (Applied Biosystem) using the following thermal cycling conditions: 15 min at 50°C (retro transcription), 2 min at 95°C (Taq polymerase activation), and 40 cycle of 15 s at 95°C and 30 s at 60°C (amplification reaction).

Target gene expression was evaluated against β-actin (β-ACT) housekeeping gene target using 2^(−ΔΔCT)^ method (26).

### Western Blotting and ELISA

Western blotting analyses were carried on culture supernatans collected after incubation of PMNs in the absence or presence of *Mmm*. PMNs supernatants were separated by NuPAGE 4–12% Bis-Tris gel (Novex, Life Technologies) at 200 V and then transferred onto iBlot2 NC stacks nitrocellulose membranes (Life Technologies) by iBlot2® Dry Blotting System (Life Technologies). Membranes were blocked with PBS containing 0.05% Tween 20 (PBST) and 5% skimmed milk for 2 h at room temperature. Membranes were incubated overnight at 4°C with specific antibodies-Rabbit anti-bovine: IL-1β (AHP851Z, Bio-Rad), 5-LOX (NB110-58749, Novus Biologicals), COX-2 (AB5118, Merck Millipore), TLR4 (A00017, Boster Biological Technology), iNOS (ADI-KAS-NO001-D, Enzo Life Sciences), and Mouse anti-bovine: IL8 polyclonal (Anti-IL8 antibody ab193818, Abcam) and monoclonal (Anti-bovine IL8 (CXCL8) mAb MT8H6, Mabtech AB) and TNFα (MCA2334, Bio-Rad). All the antibodies were diluted 1:1000 in PBST containing 2.5% skimmed milk.

After washing with PBST, membranes were incubated for 1 h at room temperature with goat anti-rabbit IgG-HRP (Bio-Rad) diluted 1:3000 or anti-mouse IgG-HRP (GE Healthcare) diluted 1:8000 in PBST containing 2.5% skimmed milk. Antigen-antibody reactions were visualized by adding chemiluminescent substrates (GE Healthcare). Images were acquired using the ChemiDoc MP (Bio-Rad) and the Image Lab Software, version 4.0.1 (Bio-Rad).

Additional tests for IL8 and TNFα were carried out on PMNs culture supernatants using commercial quantitative ELISA tests (Bovine interleukin 8 ELISA kit, MBS008105; Bovine tumor necrosis alpha ELISA kit, MBS4502967 MyBiosource) following manufacturer instructions.

### Statistical Analyses

Statistical analyses were performed applying a *t*-test for a sample (unilateral test compared to a theoretical mean of 1). The test was considered significant when observed mean was >1 with a *P* < 0.05.

## Results

Data obtained by RT-qPCR and Western blotting indicate that gene and protein expression levels of pro-inflammatory factors are modified following PMNs exposure to *Mmm*.

**Figure 1:**
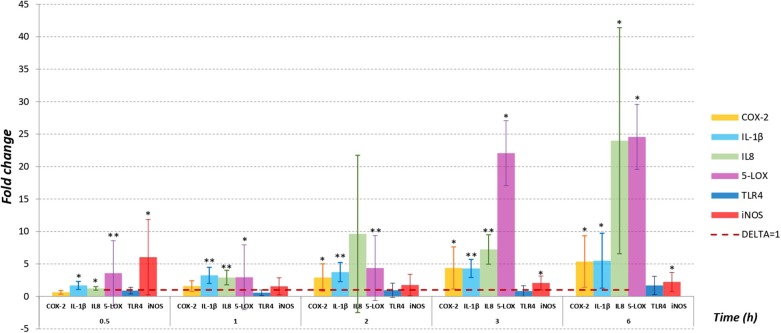
PMNs were infected with *Mmm* Caprivi at MOI = 100. RT-qPCR was applied to examine the mRNA levels and fold changes were calculated by 2^−(ΔΔCT)^ method as compared to unexposed control cells. Endogenous β-actin mRNA level was used for normalization. Fold changes were expressed as mean ± SD from three sets of independent experiments. No mRNA expression for TNFα was recorded in exposed PMNs and in control cells. No signals were appreciated at 18 h time sampling for all targeting genes. ^*^P < 0.05; ^**^P < 0.01.

Figure 1 shows the fold change in mRNA levels quantified by RT-qPCR (normalized to β-ACT) after PMNs exposure to *Mmm* for all considered time points, except for 18 h where signals for all targeting genes and for all samples (exposed PMNs and not exposed PMNs) were not appreciated.

IL-1β, IL8, and 5-LOX gene expression levels in PMNs exposed to *Mmm* were significantly higher to control values (*P* < 0.05) in all time point tested. Moreover, mRNA expression levels in treated PMNs increased in a time-dependent manner in all tested time points, with highest values observed for IL8 and 5-LOX at 3 (IL8 at 7-fold, 5-LOX at 22-fold, *P* < 0.5) and 6 h (IL8 at 24-fold, 5-LOX at 24.5-fold, *P* < 0.05) *post* PMNs treatment. Also, COX-2 mRNA expression level increased over time, but the fold changes values are significantly different (*P* < 0.05) starting from 2 h after PMNs exposure to *Mmm*.

Instead, iNOS showed a different expression profile. In fact, iNOS displayed the highest mRNA level (6-fold) at the first time point considered (30 min) while no differences to control values (*P* > 0.05) were observed at 1 h and 2 h after *Mmm* exposure.

For all time points tested by RT-qPCR, the differences observed in TLR4 expression levels were not significantly different (*P* > 0.05) compared to control values.

**Figure 2:**
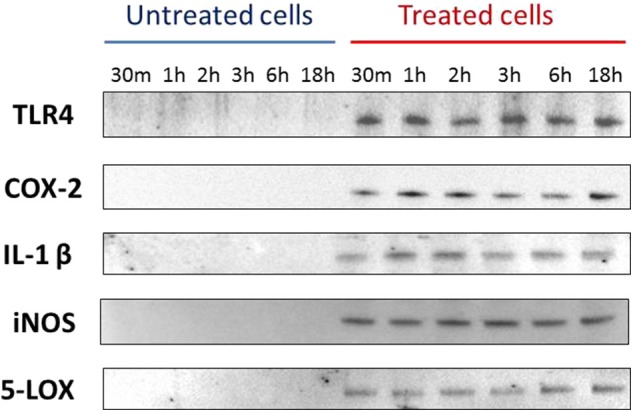
Western blotting data for TLR4, COX-2, IL-1β, iNOS, 5-LOX. Data are representative of three independent experiment.

Western blotting detected the presence of IL-1β, TLR4, iNOS, COX-2, and 5-LOX proteins, for all considered time points, starting from 30 min, only in PMNs culture supernatants of samples exposed to *Mmm* (Figure 2).

Conversely, no evidence of IL8 was detected in Western blotting while a concentration of ~100 pg/ml was detected by quantitative ELISA in both *Mmm* exposed and control samples (data not shown).

Finally, no expression for TNFα was recorded by Western blotting, ELISA and RT-qPCR assays in any of the time point tested.

## Discussion

Although investigated for long time, the pathogenesis of CBPP remains mostly unknown. This is due to several reasons including difficulties and costs to reproduce the disease experimentally in the natural host and the lack of appropriate laboratory animal models (27). Recently, *ex vivo* models based on bovine respiratory explants were developed, providing additional knowledge about host-specificity of *Mmm* (28) and its selective tropism for lower respiratory airways (bronchioles and alveoli) which represent the primary infection site (27). Acute-subacute CBPP pulmonary lesions from infected animals are characterized by a massive infiltration of inflammatory cells, with a relevant component represented by neutrophil granulocytes, together with the presence of high levels of some pro-inflammatory factors (TNFα, IL-1β, and IL-17A) (29). However, despite the clear inflammatory picture associated to CBPP lesions, transcriptomic analysis of blood samples collected from CBPP affected cattle showed that genes involved in inflammation mechanisms (as TNFα) were not upregulated during the infection (30). These findings highlight how the peripheral condition observed in blood does not necessarily reflect local inflammation, confirming the complexity of CBPP pathogenesis in which different cell types contribute to the disease development and progression. Taking this into consideration, the use of simplified *in vitro* models may contribute to dissect and clarify CBPP pathogenic mechanisms. In this research, the effects of the early interaction between *Mmm* and bovine PMNs were investigated *in vitro*, for the first time in terms of gene and protein expression, providing additional information on the involvement of these cells in the lung inflammatory response, typically observed during CBPP infection.

The obtained data indicate that *Mmm* is able to promote PMNs response *in vitro*, modulating the expression of some pro-inflammatory cytokines and inflammatory mediators released by those cells.

IL-1β and TNFα are expressed rapidly during the first stage of inflammatory response and are crucial to orchestrate a systemic and local signaling network able to promote the recognition of tissue damage (31, 32). IL-1β is expressed by different cell types through stimulation of TLRs and CD14, PMNs included, and it contributes to promote proliferation of T and B lymphocytes, activate Natural Killer cells, and stimulate the production of other inflammatory mediators including COX-2 and 5-LOX (33, 34). Data obtained in the current study indicate that *Mmm* induces a significant and progressive increase of IL-1β expression in PMNs upon to 30 min *post* exposure, supporting previous studies where high levels of IL-1β were observed in CBPP lung lesions (8, 29).

TNFα is also produced by different cells in response to TLRs stimulation, including PMNs. This cytokine is an important factor involved in the inflammatory process, regulating the expression of adhesion molecules and inducing dendritic cell maturation and chemokines production (32). The presence of TNFα in bovine affected by CBPP was demonstrated both in plasma and pulmonary lesions of CBPP infected animals (8, 29) but in this study PMNs exposed to *Mmm* did not express TNFα in terms of gene and protein expression. In our opinion the lack of TNFα expression may be due to the absence within the *in vitro* model tested of other important immune cells involved in TNFα signaling such as alveolar macrophages that represent the main source of this cytokine (32).

TLR4 is generally associated to the recognition of lipopolysaccharide antigen (LPS), but it has also been reported to be involved in mycoplasma infections (35), triggering the production of pro-inflammatory mediators involved in the immune response to bacterial infection (36). Data obtained by RT-qPCR showed no significant differences for TLR4 mRNA level between the two considered conditions (PMNs exposed to *Mmm* and PMNs not exposed) in all time point tested despite Western blotting revealed the protein expression of TLR4 only in PMNs exposed to *Mmm*. This may be due to the lysis of PMNs induced by *Mmm* exposure, causing the release of TLR4 in the treated cells supernatant.

*Mmm* induced a relevant, progressive and significant increase of IL8 expression—a chemotactic factor involved in the recruitment step—that reached its maximum pick at 6 h *post*-infection. IL8 can be produced by different cell types, comprising neutrophils, confirming that PMNs products trigger different immune cell types, comprising neutrophils themselves (37). This means that under *Mmm* stimulation, PMNs are induced to recruit other PMNs, causing an exacerbation of the inflammatory reaction. In addition to chemotaxis, IL8 also induces the production of reactive oxygen species (ROS) (38), which are suggested to play a role in the pathogenesis of CBPP (4, 7). However, at least in the *in vitro* condition tested, the amount of IL8 secreted by PMNs in culture medium did not correlate with the increased IL8 mRNA level observed. The poor correlation of IL8 protein abundance in the supernatant with high IL8 mRNA expression level may be due to post-transcriptional mechanisms that could be related to the experiment set up (39).

Although less effective than ROS, nitric oxide (NO) is a highly reactive product of nitric oxide oxidation (40) and represents an effector molecule and key mediator of non-specific immunity (41). NO is released by stimulated neutrophils in order to protect the host from harmful microorganisms and in this study, its production was very rapid. In fact, iNOS, the inducible form of the enzyme nitric oxide synthase, was the gene target more expressed by neutrophils after 30 min *post Mmm* exposure while its mRNA level decreased just after 1 h *post*-treatment.

Similarity to IL8, 5-LOX mRNA expression levels increased in a time dependent manner, reaching the highest fold change values at 3 and 6 h *post* PMNs exposure to *Mmm*. Likewise, the expression of COX-2 increased over the time, even if less pronounced then 5-LOX. COX-2 and 5-LOX are key enzymes in arachidonic acid (AA) metabolism, mediating the production of eicosanoids (42, 43). Data obtained in this study suggest that *Mmm* is able to modulate COX-2 and 5-LOX pathways, inducing the release of eicosanoid which are effective autocrine and paracrine bioactive mediators promoting the inflammatory cascade (44). Both COX-2 and 5-LOX have just been demonstrated to be involved in other mycoplasma lung infection (45) but never reported for CBPP. In fact, data showed in this study represent the first report describing the involvement of COX-2 and 5-LOX in CBPP pathogenic mechanism.

In conclusion, the achieved data indicate that *Mmm* is able to induce an early PMNs *in vitro* response in terms of gene and protein expression of some inflammatory mediators, supporting the hypothesis that *Mmm* exerts its pathogenic activity by modulating host immune response. In this case, *Mmm* directly induces PMNs activation, upregulating some pro-inflammatory mediators, such as IL-1β, IL8, 5-LOX, COX-2, and iNOS, that directly and indirectly contribute to amplify the immune and inflammatory responses taking place during CBPP infection and that may result in host tissue damage. Similar mechanisms of direct cell activation by *Mmm* could be investigated for other cells such as alveolar macrophages, endothelial cells or pneumocytes and bronchial epithelial cells, which support PMNs recruitment and probably are involved in the early stages of CBPP infection.

## Data Availability Statement

The raw data supporting the conclusions of this article will be made available by the authors, without undue reservation, to any qualified researcher.

## Author Contributions

MD and MA planned and conducted the experiments, performed RT-qPCR analysis, and wrote the manuscript. VD gave technical support for RT-qPCR analysis. IK, TD, and ML conducted the immunoblotting analysis, discussed the result, and revised the manuscript. GO contributed to antigen preparation. CC, FS, GM, MS, and MM co-supervised the work, discussed the results, and revised the manuscript.

### Conflict of Interest

The authors declare that the research was conducted in the absence of any commercial or financial relationships that could be construed as a potential conflict of interest.
